# Defining the volume of consultations for musculoskeletal infection encountered by pediatric orthopaedic services in the United States

**DOI:** 10.1371/journal.pone.0234055

**Published:** 2020-06-04

**Authors:** Jonathan G. Schoenecker, The Children’s Orthopaedic Trauma and Infection Consortium for Evidence Based Study (CORTICES) Group

**Affiliations:** Vanderbilt University Medical Center, Monroe Carell Jr. Children’s Hospital, Nashville, TN, United States of America; Department of Health, Hong Kong, HONG KONG

## Abstract

**Objective:**

Adequate resources are required to rapidly diagnose and treat pediatric musculoskeletal infection (MSKI). The workload MSKI consults contribute to pediatric orthopaedic services is unknown as prior epidemiologic studies are variable and negative work-ups are not included in national discharge databases. The hypothesis was tested that MSKI consults constitute a substantial volume of total consultations for pediatric orthopaedic services across the United States.

**Study design:**

Eighteen institutions from the Children’s ORthopaedic Trauma and Infection Consortium for Evidence-based Study (CORTICES) group retrospectively reviewed a minimum of 1 year of hospital data, reporting the total number of surgeons, total consultations, and MSKI-related consultations. Consultations were classified by the location of consultation (emergency department or inpatient). Culture positivity rate and pathogens were also reported.

**Results:**

87,449 total orthopaedic consultations and 7,814 MSKI-related consultations performed by 229 pediatric orthopaedic surgeons were reviewed. There was an average of 13 orthopaedic surgeons per site each performing an average of 154 consultations per year. On average, 9% of consultations were MSKI related and 37% of these consults yielded positive cultures. Finally, a weak inverse monotonic relationship was noted between percent culture positivity and percent of total orthopedic consults for MSKI.

**Conclusion:**

At large, academic pediatric tertiary care centers, pediatric orthopaedic services consult on an average of ~3,000 ‘rule-out’ MSKI cases annually. These patients account for nearly 1 in 10 orthopaedic consultations, of which 1 in 3 are culture positive. Considering that 2 in 3 consultations were culture negative, estimating resources required for pediatric orthopaedic consult services to work up and treat children based on culture positive administrative discharge data underestimates clinical need. Finally, ascertainment bias must be considered when comparing differences in culture rates from different institution’s pediatric orthopaedics services, given the variability in when orthopaedic physicians become involved in a MSKI workup.

## Introduction

Children diagnosed with musculoskeletal infection (MSKI) are vulnerable to significant morbidity. Prompt diagnosis and treatment with antibiotics, with or without surgical irrigation and debridement, is essential to promote optimal patient outcomes and reduce complication rates [1, 2]. Unlike other emergent consultations, such as fracture care, the heterogeneity and uncertainty upon presentation for MSKI often necessitates a considerably greater amount of clinical time and resources to accurately diagnose and treat. Furthermore, the variance in causative pathogens associated with MSKI adds complexity to the selection of the appropriate medical treatment and empiric antibiotic therapy.

To aid in the evaluation of children who are suspected to have MSKI, several retrospective studies have been conducted to estimate the volume of children who experience infection each year and evaluate clinical trends in the local epidemiology of disease [3–11]. Many of these studies have been based on administrative data and discharge diagnoses the coding of which may underestimate the total efforts devoted to rule-out infections in children who present with similar symptoms, yet ultimately receive an alternative diagnosis. Furthermore, retrospective studies have been utilized to establish diagnostic tools and clinical practice guidelines (CPGs); yet, many of these studies are focused on a single diagnosis and/or represent data from a single institution. As such, given the variation in bacterial isolates and antibiotic resistance reported between institutions [5, 6, 11, 12], the generalizability of these guidelines to the broader pediatric or infectious disease community may be limited [13–15].

To provide the pediatric community with a broader nationwide assessment of MSKI, the Children’s ORthopaedic Trauma and Infection Consortium for Evidence-based Study (CORTICES, www.cortices.org) group empirically examined the volume of pediatric orthopaedic consults, the percentage of these consults devoted to suspected MSKI, the rate of culture positivity from these consultations, and the confirmed clinical isolates at 18 tertiary pediatric centers distributed regionally throughout the United States. We hypothesized that 1) MSKIs would account for a substantial percentage of pediatric orthopaedic consultations, and 2) institutional variation exists with respect to MSKI case volume, culture positivity rate, and the spectrum of clinical isolates. By first examining national, regional, and institutional incidence and variance, the study sought to generate data that will inform pattern recognition and assist in the development of clinical MSKI guidelines that can be more broadly applicable across institutions and pediatric providers.

## Methods

Following Institutional Review Board approval from Vanderbilt University Medical Center (#151854) and completion of data use transfer agreements from each institution, a multicenter retrospective review of medical records was performed at 18 participating institutions in the CORTICES Group. This group consists of pediatric orthopaedic surgeon principal investigators at university-affiliated and stand-alone children’s hospital systems across the United States. Each institution coordinated with their hospital to obtain consult data through the EMR, emergency department records, billing records, procedure reports, and other administrative documentation for a minimum of 1-year between January 1, 2010 to December 31, 2016.

From each institution, the number of departmental pediatric orthopaedic surgeons who participate in the call schedule and received emergency department or MSKI-related consultations, the total number of orthopaedic consultations of any type, and the number of MSKI-related consultations were recorded. Patients were excluded if they experienced a penetrating trauma with a foreign body, an open-fracture, or post-surgical infection. Additionally, patients who presented with sickle cell disease, were immunocompromised, or were experiencing chronic disease state (i.e. chronic osteomyelitis) were excluded. Of patients consulted for suspected MSKI, information obtained included location where the consultation was requested (inpatient versus emergency department) and whether cultures obtained (blood and/or tissue) as part of the MSKI-related consultations were positive. To optimize accuracy, cultures were considered to be positive if blood and/or tissue cultures were positive during the admission in which the orthopaedic team was consulted. Clinical isolates detected were reported as Methicillin-sensitive *Staphylococcus aureus* (MSSA), Methicillin-resistant *Staphylococcus aureus* (MRSA), *Streptococcus pyogenes*, *Kingella kingae*, *Borrelia burgdorferi* (Lyme disease), or “other” for less commonly encountered pathogens such as *Enterobacter cloacae*, *Haemophilus influenzae*, *Pseudomonas aeruginosa*, etc.

### Data analysis and statistical examination

Institutions provided a minimum of 1 year of data based on individual analysis of data integrity based on the electronic medical record at their individual institutions. All data collected was entered in a standardized format into a REDCap database that was maintained in a deidentified, HIPPA-compliant manner at the coordinating institution (Vanderbilt University Medical Center) and adjusted on per annum values for the purpose of statistical analysis. Given that each record was fully anonymized, the IRB ethics committee waved the requirement for informed consent. To bolster data integrity, each institution had a representative present at three national CORTICES group meetings, during the course of this study, during which each CORTICES institution discussed their strategy for obtaining the relevant data. Additionally, secondary data monitoring was carried out at a central site by two independent reviewers, and resolution by discussion between the primary site coordinators and each study site was undertaken.

The number of orthopaedic consultations placed (including the distribution of consultation location, between emergency department and inpatient), the total number of MSKI-related consultations, the number of positive cultures from all MSKI-consultations, and culture and/or specialized diagnostic testing results were summarized across all sites, by region, and per institution. Pairwise comparisons across the 4 major geographic regions (Northeast, Midwest, South, and West) were conducted using chi-squared tests. P-values less than 0.05 were considered significant. All statistics were calculated using R version 3.5.2 [16].

## Results

### Orthopaedic consultation volume

Eighteen institutions completed data collection, totaling 87,449 total orthopaedic consultations and 7,814 MSKI-related consultations reviewed during the study period. On average, there were 1,957 (range: 749 to 5,173) annual orthopaedic consultations and an average of 13 (range: 4 to 25) orthopaedic surgeons per institution (**Table 1**). Of the 18 sites, 12 provided the location of the initial pediatric orthopaedic consultation (emergency department or inpatient). At these 12 sites, 68% (range: 12% to 92%) of all pediatric orthopaedic consultations were evaluated in the emergency department (**Table 1**). When comparison was made between regions, significant differences in the total number of orthopaedic consultations were observed (p<0.001) with the northeast reporting the greatest number of total pediatric orthopaedic consults; yet when normalized to the number of surgeons per institution, no significant differences in total case load per surgeon was noted between regions (p = 0.829).

**Table 1:** Annual pediatric orthopaedics consultation volume, proportion of consultations for musculoskeletal infection, and culture positivity per site.

									Culture Positive MSKI	*Regional Averages (Range)*	
	Region	Surgeons	Total Orthopaedic Consults*	Total Consults/ Surgeon*	% ED Total Consults	Total MSKI Consults*	% MSKI Consults	MSKI Consults/ Surgeon*		*% MSKI Consults*	*% Positive MSKI Culture*
**1**	**Northeast**	15	5173	345	77%	228	4.4%	15	54.5%	**4.10%**	**50.20%**
**2**	**Northeast**	19	2421	127	43%	93	3.8%	5	45.9%	**(3.8–4.4%)**	**(45.9–54.5%)**
**3**	**Midwest**	12	2208	184	*n/a*	162	7.3%	14	53.7%	**9.90%**	**46.10%**
**4**	**Midwest**	10	1593	159	*n/a*	132	8.3%	13	40.7%	**(4.3–17.9%)**	**(18.5–63.6%)**
**5**	**Midwest**	9	1478	164	68%	185	12.5%	21	60.1%		
**6**	**Midwest**	8	1075	134	*n/a*	98	9.1%	12	63.6%		
**7**	**Midwest**	23	824	36	12%	147	17.9%	6	18.5%		
**8**	**Midwest**	4	749	187	86%	33	4.3%	8	40.0%		
**9**	**South**	17	3018	178	*n/a*	360	11.9%	21	13.6%	**10.80%**	**29.70%**
**10**	**South**	25	2073	83	*n/a*	242	11.7%	10	39.7%	**(9.6–11.9%)**	**(13.6–40.0%)**
**11**	**South**	6	1977	330	92%	214	10.8%	36	31.6%		
**12**	**South**	9	1841	205	72%	183	9.9%	20	40.0%		
**13**	**South**	15	1599	107	87%	153	9.6%	10	19.6%		
**14**	**South**	6	1103	184	*n/a*	120	10.8%	20	33.9%		
**15**	**West**	10	4004	400	77%	364	9.1%	36	35.0%	**8.80%**	**28.90%**
**16**	**West**	18	1639	91	39%	163	9.9%	9	37.1%	**(7.0–9.0%)**	**(7.7–37.1%)**
**17**	**West**	13	1406	108	84%	129	9.2%	10	7.7%		
**18**	**West**	10	1052	105	80%	74	7.0%	7	35.8%		
**TOTAL**	**Average**	**13**	**1957**	**174**	**68%**	**171**	**9%**	**15**	**37.30%**	**P<0.001**	**P = 0.03**
	**SD**	**5.7**	**1104**	**94**	**23%**	**85**	**3%**	**9**	**15%**		
											*Annual

### MSKI-related consultation volume and proportion of culture positivity

Across institutions, MSKI-related consultations accounted for an average of 9.3% (range: 3.8% to 17.9%) of all orthopaedic consultations (**Table 1**). Each year, there were an average of 171 (range: 33 to 364) pediatric MSKI consultations per site. Cultures were positive in 1 out of 3 (37.3%) of pediatric MSKI consultations (range: 7.7% to 63.6%) (**Table 1**), with no significant difference in rate of culture positivity between the location of initial consultation (emergency department or inpatient) (p<0.117).

While the total number of MSKI consults annually was not statistically different across regions (average: 171 cases, range: 33 to 364) and no significant differences in MSKI-consultation load per surgeon was noted between regions (p = 0.466, average: 15 MSKI consultations per surgeon, range: 5–36 MSKI consultations), we did observe a significant difference in the proportion of MSKI consultation to total orthopaedic consultation (%MSKI consultation, p<0.001) and the percent of positive cultures detected (p = 0.03) across regions (**Table 1**), with the Northeast and the Midwest reporting higher rates of culture positivity at the time of orthopaedic consultation. The greatest proportion of MSKI consultations by orthopaedics were seen in the South (South vs all other regions: p = 0.034). The Midwest had the widest intra-region range for proportion of pediatric MSKI-related consultations to orthopaedics. No linear correlation was observed between consultation volume and rate of culture positivity (R^2^ = 0.039). However, a weak inverse monotonic relationship was noted between (Spearman’s rho -0.432, p value 0.074) percent culture positivity and percent of total orthopedic consults for MSKI.

### Causative pathogen proportions across all institutions

**Fig 1:**
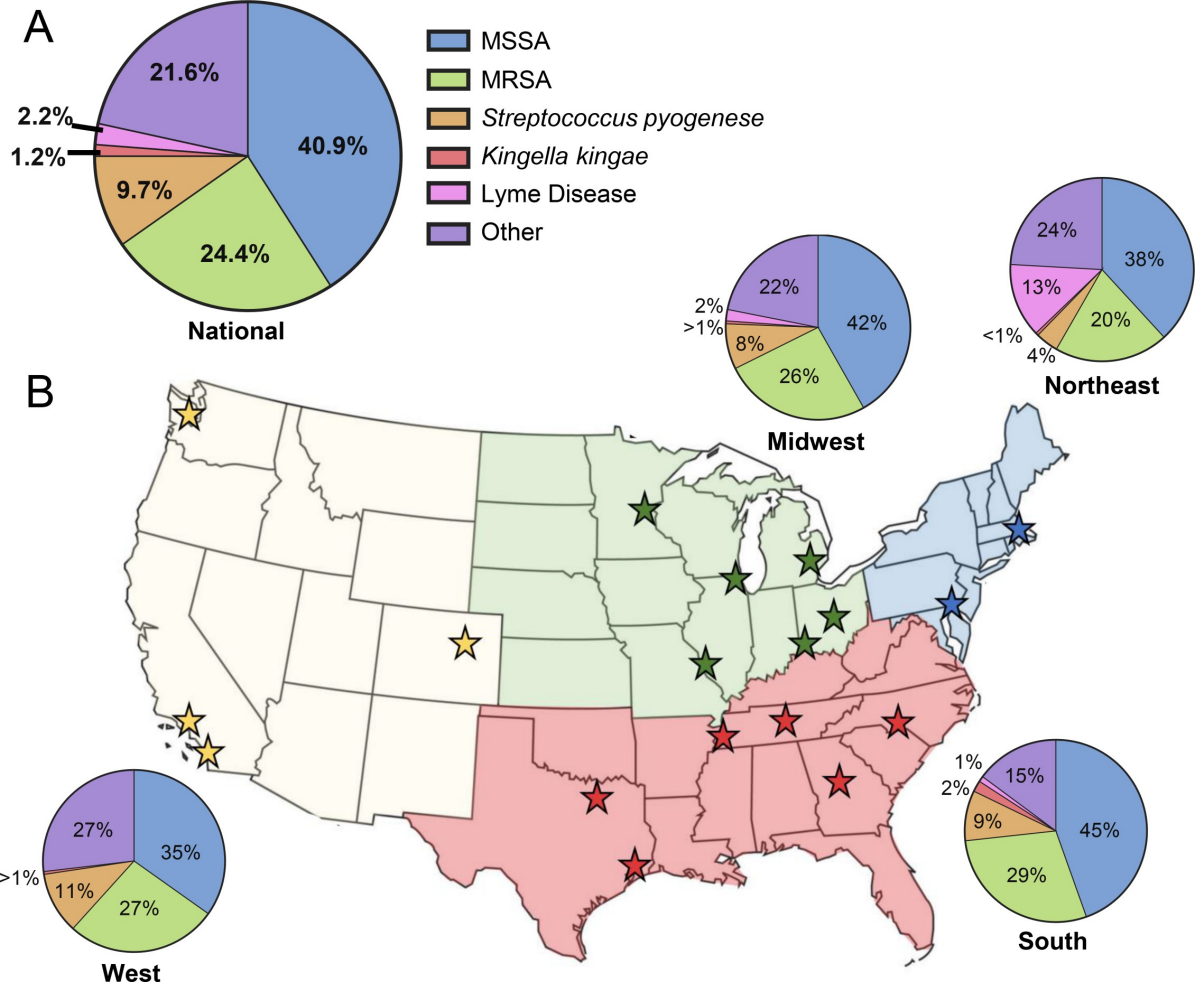
Causative pathogen proportions. A) Culture-positive results from orthopaedic consultations for MSKI across all 18 CORTICES sites. B) Proportion of positive cultures by region: West (n = 4), Midwest (n = 6), South (n = 6), and Northeast (n = 2). This figure is similar but not identical to the original image and is therefore for illustrative purposes only.

Across 18 institutions, the most common bacterial pathogen reported at the time of consultation was *Staphylococcus aureus* which accounted for ~65% of all culture-positive infections consulted and treated by pediatric orthopaedic providers of which 37.4% of confirmed staphylococcal infections were MRSA (**Fig 1A**). Culture positivity for “other” bacteria identified at the time of consultation were the second most common findings across all institutions (21.6%), followed by Group A Streptococcus (9.7%) (**Fig 1A**).

### Variation in causative pathogen proportions across regions and between institutions

When assessed by geographical region, variation was observed in the proportion of causative pathogens identified at the time of consultation, not only across regions, but also between sites within the same region (**Fig 1B and Table 2**). While Staphylococcus remained the primary cause of culture-positive MSKI across all regions, we observed a significantly higher total Staphylococcus *aureus* rate (MSSA + MRSA) in the South at the time of consultation, compared to all other regions (p = 0.039). Additionally, while not statistically significant, MRSA positive cultures were greatest in the South, with variability being seen between institutions with proportions ranging from 13% to 42% of positive cultures (**Table 2**). Comparable variability in the proportion of MRSA was observed in the West (9–50%) and Midwest (18–39%), while reports from the Northeast were consistent (20%). While inter-institutional variability was detected within regions, the average proportion of MRSA between regions was less drastic, ranging from 20–29%. Aligning with this finding, no significant differences in the ratio of MSSA:MRSA were found between regions (p = 0.969), yet we did observe variation between sites (**Table 2**).

**Table 2:** Annual percent incidence of causative pathogen proportions per site.

Site	Region	% MSSA	% MRSA	% *Streptococcus Pyogenes*	% *Kingella Kingae*	% Lyme	% Other	Ratio of MSSA/MRSA
**1**	Northeast	38%	20%	0%	1%	9%	32%	1.91
**2**	Northeast	39%	20%	8%	0%	17%	16%	1.89
**3**	Midwest	31%	39%	8%	0%	0%	22%	0.79
**4**	Midwest	35%	25%	4%	0%	0%	36%	1.37
**5**	Midwest	39%	31%	0%	1%	0%	30%	1.23
**6**	Midwest	47%	18%	13%	2%	7%	13%	2.67
**7**	Midwest	46%	21%	13%	0%	3%	18%	2.14
**8**	Midwest	52%	21%	10%	0%	0%	17%	2.44
**9**	South	36%	29%	4%	7%	0%	24%	1.26
**10**	South	32%	42%	5%	0%	0%	21%	0.78
**11**	South	36%	36%	7%	5%	2%	14%	1.00
**12**	South	56%	13%	20%	0%	0%	11%	4.18
**13**	South	57%	30%	3%	0%	0%	10%	1.89
**14**	South	51%	26%	12%	0%	1%	10%	1.96
**15**	West	40%	28%	25%	2%	0%	5%	1.43
**16**	West	35%	9%	4%	0%	0%	51%	3.77
**17**	West	27%	50%	7%	0%	0%	17%	0.53
**18**	West	38%	19%	9%	0%	0%	34%	2.00
**Comparison between Regions**		P = 0.654, ns	P = 0.590, ns	P = 0.767, ns	P = 0.970, ns	P = 0.022,*	P = 0.451, ns	P = 0.969, ns

*Streptococcus pyogenes* was observed at fairly equal rates across regions (4–11%), with the greatest proportion being found in the West, where a single institution reported that 25% of annual consults were positive for *Streptococcus pyogenes*. Likewise, the proportion of *Kingella kingae* observed was fairly equal across all regions (<1–2%), with the greatest proportion being found in the South, where a single institution reported a rate of 7%. Finally, as previously reported [17], we observed that Lyme disease was primarily seen in the Northeast, and at select sites in the Midwest (Northeast vs all other regions: p = 0.007) (**Table 2**).

## Discussion

Pediatric MSKIs generate a substantial cumulative workload for pediatric providers in the United States. Aligning with their complexity, diagnosis and treatment of pediatric MSKI requires more resources (laboratory tests, advanced imaging studies, surgical procedures), possesses the potential for multiple surgeries or readmission, and can necessitate the child to have a greater length of hospital stay [18]. This current study found that pediatric MSKI patients represent a significant percentage of patients requiring diagnostic workups and care by pediatric orthopaedic providers. Specifically, this study found that MSKI account for nearly 1 in 10 pediatric orthopaedic consultations. However, only 1 in 3 pediatric MSKI-related consultations on average produced a culture-positive identification of a clinical isolate at the time of orthopaedic consultation; further adding to the challenge of establishing a timely and accurate diagnosis.

While many prior studies have reported culture positivity rates for patients with confirmed MSKI, ranging between ~40–80% [19–21], this study more broadly assessed the culture results from all pediatric orthopaedic consultations for suspected MSKI. Thus, this assessment likewise included patients who were ultimately ruled-out for MSKI, better reflecting the clinical burden and culture success treating physicians encounter. The variance in culture positivity at the time of orthopaedic consultation, rather than indicating that a site is utilizing a superior culture strategy, may instead reflect a variance in clinical practice patterns for when orthopaedics is consulted. This is suggested by the weak inverse monotonic relationship between percent culture positivity and percent of total orthopedic consults for MSKI. Though not statistically significant, this relationship suggests that orthopaedics services with greater rates of culture positivity may be consulted later in a patient’s care, once an infection is higher on the referring physician’s differential diagnosis. This unavoidable variance in practice pattern can therefore result in ascertainment bias and the false perception of improved culture techniques; however, if all services across the pediatric hospital were considered, we would anticipate comparable culture rates between institutions.

Furthermore, the clinical indication for when orthopaedics is consulted can impact the perceived incidence of MSKI. For example, at sites where orthopaedics is consulted early in a patient’s care for a rule-out MSKI, an elevated proportion of total consults for MSKI would arise. Importantly though, this does not necessarily indicate that a site/region has a greater rate of infection, given that a portion of all consults are negative. Yet, while clinical practice may vary, the elevated incidence of consultations for MSKI still reflects a greater clinical burden on the pediatric orthopaedic service and need for resources. Thus, when developing a CPG for pediatric MSKI that spans multiple institutions, consideration must be given to the variance in practice patterns and involvement of pediatric orthopaedic services. Future studies are warranted to determine if the timing of orthopaedic consultation influences a patient length of stay, complication rate, or cost of care.

The data provided by this study broadly illustrates that the spectrum of culture positive organisms identified upon consultation is comparable between regions, with the exception of Lyme disease [17]. Aligning with prior reports [22, 23], *Staphylococcus aureus* was the most common pathogen encountered. A more detailed analysis however illustrates that the proportion of MRSA to MSSA varied widely between institutions. This is an important finding given that MRSA, in addition to increasing in prevalence and virulence over the last 10 years, has been associated with a higher rate of complication and greater severity of illness [24–27]. Furthermore, this variability calls into question the generalizability of prior clinical prediction algorithms used to predict MRSA from MSSA in children [25]. Given that such algorithms have been built from data of single institutions, this may explain the poor predictive capability when used at an institution with a different incidence of MRSA [28]. From this nationwide assessment, we observed an average MSSA:MRSA ratio of 1.84 at the time of consultation across all institutions. This information can be used as a benchmark value for the anticipated rate of MRSA and MSSA, such that if an institution is experiencing a markedly greater ratio of MRSA at the time of consultation, this may indicate the need for an intervention to address a disproportionately higher relative rate. Finally, although rarer, this study likewise found variance in the reported proportions of *Kingella kingae* between sites. This result may be due to a true elevated incidence, or ascertainment bias due to variance in diagnostic testing at these institutions due to availability, knowledge of culture, and/or 16S PCR-based assay. Therefore, when developing CPGs for pediatric MSKI, in addition to pathogen variance, clinical workflow and diagnostic capacity must also be considered for the multiple services involved in the diagnosis and care of pediatric MSKIs.

Much of the current literature on pediatric MSKI has been focused specifically on diagnosis and treatment algorithms, without defining the overall volume of MSKIs [13, 29–33]. Prior studies have attempted to classify pediatric MSKI by level of severity rather than diagnosis [11, 12, 32–34]. These studies, however, have not defined how frequently these cases are encountered by pediatric orthopaedic surgeons. While large national discharge databases, such as the Pediatric Hospital Information System and the Kids’ Inpatient Database (https://www.hcup-us.ahrq.gov/db/nation/kid/kiddbdocumentation.jsp), contain a large volume of data regarding procedure and diagnosis codes, they fail to account for the chronology of events associated with a particular hospitalization, making this type of analysis impossible. Furthermore, while administrative databases can provide broad data sets which are potentially population based, their accuracy has been called into question, particularly in the setting of diagnoses, outcomes, and complications [35]. This may be of particular concern in the setting of MSKI, given that workups can be negative or equivocal, thereby excluding these consultations in the discharge databases and leading to potential misclassification bias of cases and controls.

To our knowledge, this is the largest, multi-center pediatric musculoskeletal infection databased to-date. Unlike the assessment of laboratory values or patient outcomes which are commonly collected from patient records in the same manner, information on consultations are not uniformly preserved across institutions and thus at each institution unique strategies were employed to search the administrative records for medical staffing, electronic medical records, human resources, or emergency department and inpatient databases to obtain accurate information. As a result, we believe the data presented within this manuscript is more accurate and reliable than many of the previously published original scientific studies or reviews related to clinical practice patterns and burden of pediatric MSKI.

### Study limitations

The results of this study are limited by the retrospective study design. In this study we did not present information on the specific diagnosis or the location of the MSKI, as the goal of this study was to determine the average volume of pediatric MSKI encountered at 18 institutions across the United States in a comprehensive manner with less bias towards severe infections. Our sites were limited to academic and stand-alone children’s hospitals that are often referral centers. Thus, this data may not be generalizable to reflect the practice patterns observed in community hospitals, given the potential for case severity bias [36, 37]. Furthermore, while we assessed the number of orthopaedic surgeons at each institution, we did not account for overlapping duties such as daytime clinic, which may compound the burden of MSKI consultations on treating orthopaedic surgeons. Data in this study was collected from various sites around the United States, yet, there was greater representation of sites in the Midwest and South regions; therefore, the results may be biased toward pediatric MSKI encountered in these regions. Nevertheless, the current data accounts for the high frequency of MSKI-related consultations which result in negative or equivocal workups, which previous studies have not well described. Such a methodological approach is critical in assessing the true volume of MSKI on a national scale and its burden on healthcare providers and the system as a whole. Finally, although the duration of data captured from each site was variable, this study represents the “best available” data given individual centers medical record deficiencies; furthermore, to systematically address this source of bias we adjusted the data per annum.

## Conclusions

A substantial number of children with suspected or confirmed MSKI are evaluated and treated by pediatric orthopaedic surgeons in the United States. Across the 18 CORTICES sites assessed, pediatric MSKI account for nearly 1 in 10 pediatric orthopaedic consultations on average, of which 1 in 3 consultations were culture-positive. Therefore, considering that nearly 2 out of 3 consultations for ‘rule-out’ MSKI in children were culture-negative, prior estimates of resources required based on culture-positive administrative discharge data will greatly underestimate current clinical need. While Staphylococcus is common across all regions and institution, we observed a variance in the proportion of MSSA to MRSA between institutions. Through the establishment of a more reliable pathogen-specific national proportions, these data can serve as a benchmark for detecting higher prevalence of virulent pathogens, such as MRSA, and the need for public health interventions. Finally, ascertainment bias must be considered when comparing differences in culture rates from different institution’s pediatric orthopaedics services given the variability in when these services become involved in a MSKI workup.

## Supporting information

S1 File (DOCX)
