# Correction: Defining the volume of consultations for musculoskeletal infection encountered by pediatric orthopaedic services in the United States

**DOI:** 10.1371/journal.pone.0237942

**Published:** 2020-08-13

**Authors:** The PLOS ONE Staff

The Children’s Orthopaedic Trauma and Infection Consortium for Evidence Based Study (CORTICES) Group authors are missing from the article byline. The publisher apologizes for the error. The correct author byline and affiliations are as follows:

Ryan J. Koehler^1^, Benjamin J. Shore^2^, Daniel Hedequest^2^, Benton E. Heyworth^2^, Collin May^2^, Patricia E. Miller^2^, Emily S. Rademacher^2^, Ryan M. Sanborn^2^, Joshua S. Murphy^3^, Alyssa Roseman^4^, Jason W. Stoneback^4^, Anastasiya A. Trizno^4^, Rachel Y. Goldstein^5^, Liam Harris^5^, Ena Nielsen^5^, Divya Talwar^6^, Jaime R. Denning^7^, Noor Saeed^7^, Brooke Kutz^8,9^, Jennifer C. Laine^8,9^, Mary Naas^8,9^, Walter H. Truong^8,9^, Matthew Rotando^10^, David D. Spence^10^, Brian K. Brighton^11^, Christine Churchill^11^, Joseph A. Janicki^12^, Kiana King^12^, Jacob Wild^12^, Allan C. Beebe^13^, Schon Crouse^13^, Teaya Rough^13^, Mallory Rowan^13^, Satbir Singh^13^, Amanda Davis-Juarez^14^, Adam Gould^14^, Olivia Hughes^14^, Kathleen D. Rickert^14^, Vidyadhar V. Upasani^14^, Todd J. Blumberg^15^, Viviana Bompadre^15^, Antoinette W. Lindberg^15^, Mark L. Miller^16^, Jaclyn F. Hill^17^, Hayley Peoples^17^, Scott B. Rosenfeld^17^, Rod Turner^17^, Lawson A. Copley^18^, Eduardo A. Lindsay^18^, Brandon A. Ramo^18^, Naureen Tareen^18^, R. Lane Wimberly^18^, G. Ying Li^19^, Jordyn Sessel^19^, Megan E. Johnson^1^, Samuel Johnson^1^, Stephanie N. Moore-Lotridge^1^, Julie Shelton^1^, Keith D. Baldwin^6^, and Jonathan G. Schoenecker^1^ on behalf of the Children’s Orthopaedic Trauma and Infection Consortium for Evidence Based Study (CORTICES) Group

**1** Vanderbilt University Medical Center, Monroe Carell Jr. Children’s Hospital, Nashville, TN, United States of America,

**2** Boston Children’s Hospital, Boston, MA, United States of America,

**3** Children’s Healthcare of Atlanta, Atlanta, GA, United States of America,

**4** Children’s Hospital Colorado, Aurora, CO, United States of America,

**5** Children’s Hospital of Los Angeles, Los Angeles, CA, United States of America,

**6** Children’s Hospital of Philadelphia, Philadelphia, PA, United States of America,

**7** Cincinnati Children’s Hospital Medical Center, Cincinnati, OH, United States of America,

**8** Gillette Children’s Specialty Healthcare, St. Paul, MN, United States of America,

**9** Children’s Minnesota, Minneapolis, MN, United States of America,

**10** Le Bonheur Children’s Hospital, Memphis, TN, United States of America,

**11** Levine Children’s Hospital, Charlotte, NC, United States of America,

**12** Lurie Children’s Hospital of Chicago, Chicago, IL, United States of America,

**13** Nationwide Children’s Hospital, Columbus, OH, United States of America,

**14** Rady Children’s Hospital, San Diego, CA, United States of America,

**15** Seattle Children’s Hospital, Seattle, WA, United States of America,

**16** St. Louis Children’s Hospital at Washington University, St. Louis, MO, United States of America,

**17** Texas Children’s Hospital, Houston, TX, United States of America,

**18** Texas Scottish Rite Hospital for Children/ Children’s Medical Center Dallas, Dallas, TX, United States of America,

**19** University of Michigan- C.S. Mott Children’s Hospital, Ann Arbor, MI, United States of America

The correct citation is: Koehler RJ, Shore BJ, Hedequest D, Heyworth BE, May C, Miller PE, et al. (2020) Defining the volume of consultations for musculoskeletal infection encountered by pediatric orthopaedic services in the United States. PLoS ONE 15(6): e0234055. https://doi.org/10.1371/journal.pone.0234055

The correct Author Contributions are as follows:

**Conceptualization: **Ryan J. Koehler, Benjamin J. Shore, Daniel Hedequist, Benton E. Heyworth, Collin May, Joshua S. Murphy, Jason W. Stoneback, Rachel Y. Goldstein, Jaime R. Denning, Jennifer C. Laine, Walter H. Truong, Brian K. Brighton, Joseph A. Janicki, Allan C. Beebe, Kathleen D. Rickert, Vidyadhar V. Upasani, Todd J. Blumberg, Viviana Bompadre, Antoinette W. Lindberg, Mark L. Miller, Jaclyn F. Hill, Scott B. Rosenfeld, Lawson A. Copley, Brandon A. Ramo, R. Lane Wimberly, G. Ying Li, Megan E. Johnson, Keith D. Baldwin, Jonathan G. Schoenecker

**Data curation:** Ryan J. Koehler, Benjamin J. Shore, Daniel Hedequist, Benton E. Heyworth, Collin May, Patricia E. Miller, Emily S. Rademacher, Ryan M. Sanborn, Joshua S. Murphy, Alyssa Roseman, Jason W. Stoneback, Anastasiya A. Trizno, Rachel Y. Goldstein, Liam Harris, Ena Nielsen, Divya Talwar, Jaime R. Denning, Noor Saeed, Brooke Kutz, Jennifer C. Laine, Mary Naas, Walter H. Truong, Matthew Rotando, Brian K. Brighton, Christine Churchill, Joseph A. Janicki, Kiana King, Jacob Wild, Allan C. Beebe, Schon Crouse, Teaya Rough, Mallory Rowan, Satbir Singh, Amanda Davis-Juarez, Adam Gould, Olivia Hughes, Kathleen D. Rickert, Vidyadhar V. Upasani, Todd J. Blumberg, Viviana Bompadre, Antoinette W. Lindberg, Mark L. Miller, Jaclyn F. Hill, Hayley Peoples, Scott B. Rosenfeld, Rod Turner, Lawson A. Copley, Eduardo A. Lindsay, Brandon A. Ramo, Naureen Tareen, R. Lane Wimberly, G. Ying Li, Jordyn Sessel, Megan E. Johnson, Samuel Johnson, Stephanie N. Moore-Lotridge, Julie Shelton, Keith D. Baldwin, Jonathan G. Schoenecker

**Formal analysis:** Ryan J. Koehler, Benjamin J. Shore, Daniel Hedequist, Benton E. Heyworth, Collin May, Patricia E. Miller, Emily S. Rademacher, Ryan M. Sanborn, Joshua S. Murphy, Alyssa Roseman, Jason W. Stoneback, Anastasiya A. Trizno, Rachel Y. Goldstein, Liam Harris, Ena Nielse, Divya Talwar, Jaime R. Denning, Noor Saeed, Brooke Kutz, Jennifer C. Laine, Mary Naas, Walter H. Truong, Matthew Rotando, Brian K. Brighton, Christine Churchill, Joseph A. Janicki, Kiana King, Jacob Wild, Allan C. Beebe, Schon Crouse, Teaya Rough, Mallory Rowan, Satbir Singh, Amanda Davis-Juarez, Adam Gould, Olivia Hughes, Kathleen D. Rickert, Vidyadhar V. Upasani, Todd J. Blumberg, Viviana Bompadre, Antoinette W. Lindberg, Mark L. Miller, Jaclyn F. Hill, Hayley Peoples, Scott B. Rosenfeld, Rod Turner, Lawson A. Copley, Eduardo A. Lindsay, Brandon A. Ramo, Naureen Tareen, R. Lane Wimberly, G. Ying Li, Jordyn Sessel, Megan E. Johnson, Samuel Johnson, Stephanie N. Moore-Lotridge, Julie Shelton, Keith D. Baldwin, Jonathan G. Schoenecker

**Funding acquisition:** Jonathan G. Schoenecker

**Project administration:** Benjamin J. Shore, Ryan M. Sanborn, Julie Shelton, Keith D. Baldwin, Jonathan G. Schoenecker

**Writing–original draft:** Ryan J. Koehler, Stephanie N. Moore-Lotridge, Jonathan G. Schoenecker

**Writing–review & editing:** Benjamin J. Shore, Daniel Hedequist, Benton E. Heyworth, Collin May, Patricia E. Miller, Emily S. Rademacher, Ryan M. Sanborn, Joshua S. Murphy, Alyssa Roseman, Jason W. Stoneback, Anastasiya A. Trizno, Rachel Y. Goldstein, Liam Harris, Ena Nielsen, Divya Talwar, Jaime R. Denning, Noor Saeed, Brooke Kutz, Jennifer C. Laine, Mary Naas, Walter H. Truong, Matthew Rotando, Brian K. Brighton, Christine Churchill, Joseph A. Janicki, Kiana King, Jacob Wild, Allan C. Beebe, Schon Crouse, Teaya Rough, Mallory Rowan, Satbir Singh, Amanda Davis-Juarez, Adam Gould, Olivia Hughes, Kathleen D. Rickert, Vidyadhar V. Upasani, Todd J. Blumberg, Viviana Bompadre, Antoinette W. Lindberg, Mark L. Miller, Jaclyn F. Hill, Hayley Peoples, Scott B. Rosenfeld, Rod Turner, Lawson A. Copley, Eduardo A. Lindsay, Brandon A. Ramo, Naureen Tareen, R. Lane Wimberly, G. Ying Li, Jordyn Sessel, Megan E. Johnson, Samuel Johnson, Stephanie N. Moore-Lotridge, Julie Shelton, Keith D. Baldwin, Jonathan G. Schoenecker
