# Supplementary material for: Defining the volume of consultations for musculoskeletal infection encountered by pediatric orthopaedic services in the United States
Source: PLoS One. 2020 Jun 4;15(6):e0234055. doi: 10.1371/journal.pone.0234055 (PMC7272072; doi:10.1371/journal.pone.0234055)
Supplement: S1 File — (DOCX) [file pone.0234055.s001.docx]

CORTICES Group Authorship:

Vanderbilt University Medical Center- Monroe Carell Jr. Children’s Hospital, Nashville, TN, USA

Ryan J. Koehler, MD; Megan E. Johnson, MD; Samuel Johnson, BS; Stephanie N. Moore-Lotridge, PhD; Julie Shelton, BAA CCRP; Jonathan G. Schoenecker, MD PhD*

Boston Children’s Hospital, Boston, MA, USA

Daniel Hedequist, MD; Benton E. Heyworth, MD; Collin May, MD MPH; Patricia E. Miller, MS; Emily S. Rademacher, BS; Ryan M. Sanborn, BA; Benjamin J. Shore, MD MPH FRCSC

Children’s Hospital of Atlanta, Atlanta, GA, USA

Joshua S. Murphy, MD

Children’s Hospital Colorado, Aurora, CO, USA

Alyssa Roseman, MSc; Jason W. Stoneback, MD; Anastasiya A. Trizno, BS

Children’s Hospital of Los Angeles, Los Angeles, CA, USA

Rachel Y. Goldstein, MD MPH; Liam Harris; Ena Nielsen

Children’s Hospital of Philadelphia, Philadelphia, PA, USA

Keith D. Baldwin, MD MPH MSPT; Divya Talwar

Cincinnati Children’s Hospital Medical Center, Cincinnati, OH, USA

Jaime R. Denning, MD; Noor Saeed

Gillette Children’s Specialty Healthcare and Children’s Minnesota, St. Paul, MN, USA

Brooke Kutz, MD; Jennifer C. Laine, MD; Mary Naas

Le Bonheur Children’s Hospital, Memphis, TN, USA

Matthew Rotando; David D. Spence, MD

Levine Children’s Hospital, Charlotte, NC, USA

Brian K. Brighton, MD MPH; Christine Churchill, MA

Lurie Children’s Hospital of Chicago, Chicago, IL, USA

Joseph A. Janicki, MD; Kiana King; Jacob Wild

Nationwide Children’s Hospital, Columbus, OH, USA

Allan C. Beebe, MD; Schon Crouse; Teaya Rough; Mallory Rowan; Satbir Singh

Rady Children’s Hospital, San Diego, CA, USA

Amanda Davis-Juarez, BS; Adam Gould, BS; Olivia Hughes, BS; Kathleen D. Rickert, MD; Vidyadhar V. Upasani, MD

Seattle Children’s Hospital, Seattle, WA, USA

Todd J. Blumberg, MD; Viviana Bompadre, PhD; Antoinette W. Lindberg, MD

St. Louis Children’s Hospital at Washington University, St. Louis, MO, USA

Mark L. Miller, MD

Texas Children’s Hospital, Houston, TX, USA

Jaclyn F. Hill, MD; Hayley Peoples; Scott B. Rosenfeld, MD; Rod Turner

Texas Scottish Rite Hospital for Children/ Children’s Medical Center Dallas, Dallas, TX, USA

Lawson A. Copley, MD; Eduardo A. Lindsay, MD; Brandon A. Ramo, MD; Anthony I. Riccio, MD; Naureen Tareen; R. Lane Wimberly, MD

University of Michigan- C.S. Mott Children’s Hospital, Ann Arbor, MI, USA

G. Ying Li, MD; Jordyn Sessel, BS
